# Diabetes related knowledge, self-care behaviours and adherence to medications among diabetic patients in Southwest Ethiopia: a cross-sectional survey

**DOI:** 10.1186/s12902-016-0114-x

**Published:** 2016-05-31

**Authors:** Tefera Kassahun, Hailay Gesesew, Lillian Mwanri, Tesfahun Eshetie

**Affiliations:** Dilchora Hospital, Diredawa, East Ethiopia; Department of Epidemiology, College of Health Sciences, Jimma University, Jimma, Ethiopia; Discipline of Public Health, Faculty of Medicine, Nursing and Health Sciences, Flinders University, Adelaide, Australia; Department of Clinical Pharmacy, College of Health Sciences, Jimma University, Jimma, Ethiopia

**Keywords:** Knowledge, Self-care behaviour, Adherence, Type 2 diabetes mellitus, Ethiopia

## Abstract

**Background:**

The provision of health education involving self-care and good adherence to medications has been acknowledged to be a cost effective strategy for improving quality of life of diabetes patients. We assessed levels of knowledge about type 2 diabetes mellitus (T2DM), self-care behaviours and adherence to medication among DM patients.

**Methods:**

A facility based cross-sectional survey of 325 adults with T2DM patients attending Jimma University Teaching Hospital, Southwest Ethiopia was conducted. We used diabetes Knowledge Test, Expanded Version of the Summary of Diabetes Self-Care Activities and Morisky 8-Item medication adherence as tools to measure diabetic knowledge, self-care behaviours and adherence to medications respectively. Multinomial logistic regression analyses were used to assess the independent predictors of diabetes knowledge and adherence to medications. The binary logistic regression was applied for self-care behaviours.

**Results:**

309 respondents were included in the survey. Of all the respondents, 44.9 %, 20.1 % and 34.9 % had low, medium and high level diabetic knowledge respectively. High level of diabetic knowledge was the reference group. Being illiterate (AOR = 3.1, 95%CI: 1.03-9.3), having BMI <18 kg/m^2^ (AOR = 6.4, 95%CI: 1.2-34.9) and duration of DM < 5 years (AOR = 4.2, 95%CI: 1.9-9.5) were significantly associated with low level of diabetic knowledge. T2DM patients who practiced good self-care (AOR = 0.5, 95%CI: 0.3-0.9) were less likely to have low knowledge. Duration of DM < 5 years (AOR = 9.8, 95%CI: 3.2-30.2) was significantly associated with medium level of diabetic knowledge. 157(50.8 %) patients had poor self-care behaviour and this was associated with level of education and adherence to medication. The proportions of patients with low, medium and high adherence to medication were 24.9 %, 37.9 % and 37.2 % respectively. Being a merchant, having medium level of diabetic knowledge and having good glycemic control level were associated with low adherence to medications.

**Conclusions:**

Significant number of DM patients had low level of knowledge, poor self-care behaviours and low level of adherence to medications. These findings call for the need of integrated interventional management on diabetic knowledge, self-care behaviours and adherence to medications. To ensure effective T2DM management, a strategic approach that improves health literacy could be a cross cutting intervention.

**Electronic supplementary material:**

The online version of this article (doi:10.1186/s12902-016-0114-x) contains supplementary material, which is available to authorized users.

## Background

Diabetes mellitus (DM) is a serious global public health problem, an illness that kills silently. In 2013, it was reported that DM killed 4.6 million people, [1] with further 387 million reported to being affected in 2014 [2]. More than 77 % of morbidity [2] and 88 % of mortality [3] resulting from DM occurred in low- and middle- income countries in 2012. Diabetes mellitus scourge is expected to affect nearly 592 million people globally by the end of 2035 [2]. In Ethiopia, the prevalence of diabetes was 3.5 % in 2011, [4] and the extrapolated prevalence in 2013 was 4.36 % [2]. Reportedly, 34,262 patients out of 1.8 million DM cases died in Ethiopia in 2013 [2]. It is also known that a large number of people remain undiagnosed, with estimated number of undiagnosed cases reported to be 1.39 million people in 2013 [2]. Type 2 diabetes mellitus (T2DM) is the most common form of DM worldwide, accounting for more than 90 % of cases [1].

Diabetes is a chronic disease significantly affecting the quality of life of affected populations and can lead to poor health outcomes of individuals, families and communities [5]. Its impact affects social and economic outcomes, [6] including costing millions of health care budgets of nations [2] across the world [7]. The risk factors for DM include raised blood pressure, tobacco use, alcohol consumption, physical inactivity, poor dietary patterns and overweight [8, 9]. Poor adherence to medications and poor self-care behaviours have also been reported to be barriers for effective management of DM complications [8, 10]. Most of the risk factors of DM and its complications are modifiable. Self-management strategies such as self-monitoring of blood glucose, dietary restrictions, regular foot care and ophthalmic examinations have all been shown to markedly reduce the incidence and progression of DM complications,[1] and these can be achieved by patients themselves via effective education and enhanced knowledge [11].

Evidences from earlier studies have supported the notion that having good knowledge and education have influence to good care and can reduce DM complications significantly [12, 13]. Knowledge not only enhances the self-care behaviours [1], but it enables DM patients to adhere to their treatment effectively. It has also been noted that age, lack of resources and perceived side effects have significant association with poor adherence to medication [8].

Knowledge, self-care behaviours and adherence to medications in diabetes could be helpful for early case detection, prevention, and minimization of complications, and improvements of the quality of life of affected individuals. Previous studies have reported poor health outcomes to be associated within insufficient knowledge, poor self-care behaviours and adherence to medications among diabetic patients [14–16]. There is insufficient work regarding knowledge, self-care behaviours and adherence to medications related to DM in Ethiopia. Studies to provide evidences for these factors are noteworthy for prevention and control of diabetes and other non-communicable diseases (NCDs), and to inform policies and strategies in such resource meager countries. We assessed the levels of knowledge, self-care behaviours and adherence to medications about diabetes mellitus among diabetic adult patients in Ethiopia.

## Methods

### Study design, settings and participants

A facility based cross-sectional study was carried out in diabetic clinic at Jimma University Teaching Hospital (JUTH), Southwest Ethiopia between February and April 2014. We followed Drug Administration and Control Authority of Ethiopia guidelines [17] for diagnosis and classification of DM. These guidelines are similar to the criteria developed by International Diabetes Federation (IDF) [18]. The study was conducted among T2DM adult patients (≥18 years) who were for at least four visits.

**Fig. 1:**
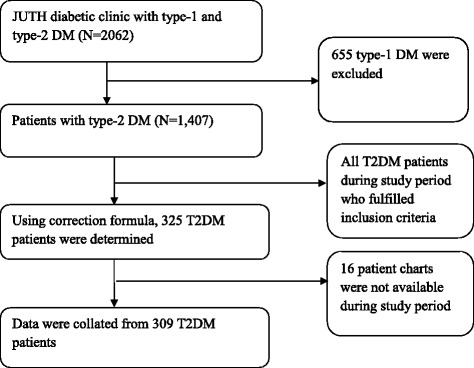
Summary of flowchart record selection, 2014

This work was conducted alongside our previously published paper on glycemic control [19]. Originally, the project had four outcomes: glycemic control, knowledge, self-care behavior and medication adherence. The sample size calculation considering all outcomes and ‘glycemic control’ gave us the maximum sample size that helped us to look at various factors that affect the outcomes. The sample size was calculated via OpenEpi 2.3 software using a single population proportion calculation formula using the following assumptions: 58.2 % proportion [8], 95 % confidence level, 5 % margin of error and 10 % non-response rate. Considering a correction formula, the total calculated sample yielded 325. Using sampling frame of DM records, simple random sampling technique was used to recruit the study participants (Fig. 1).

### Dependent variables

Diabetes knowledge, self-care behaviours and adherence to medications were the dependent variables. Diabetes Knowledge was measured using The Diabetes Knowledge Test (DKT) [20, 21]. The DKT was developed and tested for reliability and validity by the University of Michigan scholars and was adapted for the Ethiopian context. The DKT is a 23-item multiple-choice test designed to assess knowledge about diet, exercise, blood glucose levels and testing and self-care activities. Each item had three or four multiple choices with only one correct answer. The first 14 items were designed for all adults with diabetes, while items 15–23 apply only to those using insulin [21]. Scores on the DKT were computed for each participant. The score was determined by dividing the number of correct answers by the total number of questions (23 questions for patients taking insulin and 14 for those receiving oral hypoglycemic agents). Scores ≥75 %, 74-60 % and ≤59 %, respectively, were labeled as high, medium and low knowledge on diabetes [20]. Internal consistency of the tools for knowledge was measured by Cronbach’s alpha and was adequate (Cronbach’s alpha =0.78).

Self-care behaviours were assessed using Expanded Version of the Summary of Diabetes Self-Care Activities (SDSCA) [22]. The SDSCA was originally developed from “The Summary of Diabetes Self-Care Activities Measure” that resulted from seven studies carried out by scholars from Oregon Research Institute, United States. The tools were adapted for the Ethiopian Context. We adapted some foodstuffs, for instance high fat diet food types, and terminologies mentioned in the original version to fit Ethiopian context. Each scale measured frequency of self-care activity in the last 7 days for the following aspects of the diabetes regimen: general diet, foot-care, exercise and medication taking. The score was presented in terms of mean number of days for each self-care behaviours, which was calculated by summation of number of days of self-care practice divided by total number of patients. The overall mean score was calculated by summation of the mean score for diet, foot-care, exercise and medication taking divided by the sum of number of questions under each scale. After calculating an overall mean score, it was classified as having good self-care behaviour if the patient scored ≥3 or poor self-care behaviour if the patient scored <3.

Adherence to medications was measured using Morisky scale [23] that was prepared from “Concurrent and predictive validity of a self-reported measures of adherence to medications”. The tool was originally developed by scholars from the University of California, University of Texas and John Hopkins Research institute, but again adapted for Ethiopian use. It consisted of 8-item questionnaire with yes or no responses coded respectively as 1 or 0. But for one question, the score was given inversely. The total score of adherence to medications was classified in to low adherence if the score was >2, medium adherence if between 1 and 2, and high adherence if 0.

### Independent variables

The explanatory variables included: socio-demographic and economic data (age, sex, education level, marital status, occupation, income, ethnicity and religion), history of smoking, history of alcohol consumption, family history of DM, duration of therapy, body mass index (BMI) and glycemic control level. Level of education was classified as illiterate (couldn’t read and write), primary (received education up to class eight), and secondary and above (received education class from grade nine and above).

History of smoking and history of alcohol consumption has been assessed as during lifetime. We asked participants to report their lifetime experience of cigarette smoking and alcohol drinking. We recorded their response as smoker, non-smoker and ex-smoker for cigarette smoking whereas for alcohol drinking we asked their status as drinker or non- drinker. For statistical analysis, we further aggregated their responses to yes or no binary responses. Family history of DM was measured if any family member (mother or father) had DM. Anthropometric measurements were used to assess the body mass index (BMI). Glycemic level was coded as poor or good. Poor glycemic control was defined if fasting blood glucose (FBG) level was above 130 mg/dl. Patients FBG reading for at least four months were recorded and the mean blood glucose level computed [24].

### Interviews

Six face-to-face interviews were conducted by six registered nurses and one public health officer to solicit socio-demographic and economic data, medication adherence, diabetic knowledge and self-care behavior of participants. The interviews were conducted in a quiet room at the DM clinic where patients came for follow up checkup. Validated and structured interviewer rater questionnaires were used for data collection. The Morisky medication adherence scale questionnaires was used to solicit participants’ adherence to taking medications, whereas Expanded Version of the Summary of Diabetes Self-Care Activities (SDSCA) Self-care behaviours questionnaire were used to assess participants self-care behavior adherence. The Diabetes Knowledge Test (DKT) tool, a 23 multiple-choice question test was used to measure participants’ diabetes knowledge. The tools were firstly designed in English and then translated in Amharic and Afan Oromo (local languages), and again back translated into English by experts who had similar experiences (Additional file 1). Questionnaires were pre-tested with diabetic patients in another nearby hospital at Jimma and necessary modifications were made.

### Statistical analysis

Descriptive statistics included mean, median, standard deviations, and range values for continuous data; percentage and frequency tables for categorical data. We used multinomial logistic regression to analyze factors that were associated with diabetes knowledge and adherence to medications. To assess factors associated with self-care behaviours, binary logistic regression analysis was used. For both multinomial and binary logistic regression analyses, bivariate and multiple regression assessment was conducted to check the existence of crude association and select the candidate variables (*P* < 0.25 was considered).

We checked multi-collinearity among selected independent variables via variance inflation factor (VIF) and none was found. *P*-value < = 0.05 was considered as a cut off point for statistical significance in the final model. Fitness of goodness of the final model was checked by Hosmer and Lemeshow and was found fit. The Data were summarized using odds ratio (OR) and 95 % confidence interval. The analyses were conducted in Statistical Package for the Social Sciences (SPSS) version 22.0 for mackintosh.

## Results

### Socio-demographic and clinical characteristics of respondents

Three hundred and twenty five (325) DM patients were considered eligible, but 16 were excluded because their charts were not available (Fig. 1). In total, 309 (95 %) patients were included in the analysis.

Table 1 shows demographic characteristics of the respondents. Males were over-represented (61.8 %) and almost two fifth (36.9 %) of the respondents represented the age group 51–60 years. Nearly half (46.6 %) of the respondents followed Muslim religion and four out of five (81.2 %) respondents were married. Two fifth (36.2 %) respondents attained grades 1–8, and 30.4 % of respondents were farmers.

**Table 1:** Frequency distributions of socio-demographic characteristics of T2DM patients on follow up at JUTH, 2014

Socio-demographic characteristics (*n* = 309)	Categories	n (%)
Sex	Male	189 (61.8)
	Female	120 (38.2)
Age	<40 years 40-60 years > = 60 years Missing	12 (3.9) 188 (60.8) 87 (28.2) 22 (7.1)
Marital status	Married	251 (81.3)
	Single	21 (6.8)
	Widow/er	37 (11.9)
Ethnicity	Oromo	170 (55.0)
	Amhara	78 (25.2)
	Keficho	21 (6.8)
	Gurage	10 (3.2)
	Dawero	8 (2.6)
	Yem	8 (2.6)
	Other^a^	14 (4.5)
Religion	Muslim	144 (46.6)
	Orthodox Protestant Others^b^	138 (44.7) 23 (7.4) 4 (1.3)
Level of education	Illiterate Primary 2^0^ and above	109 (35.3) 112 (36.3) 88 (28.4)
Occupation	Employed	72 (23.3)
	Unemployed	78 (25.2)
	Merchant	29 (9.4)
	Farmer	94 (30.4)
	Daily labor	36 (11.7)

^a^Tigre, wolayita, ^b^Catholic, Jehovah witness

*T2DM* type 2 diabetes mellitus, *JUTH* Jimma University Teaching Hospital

### Level of knowledge on diabetes

Table 2 shows the distribution of subjects’ knowledge of diabetes by demographic and clinical characteristics. Among the patients, 44.9 %, 20.1 % and 34.9 % had low, medium and high level of knowledge of diabetes respectively. For respondents who had low level of knowledge of diabetes, 30.9 % had low adherence to medications, 56.1 % had poor self-care behaviours and 28.1 % had poor glycemic control level. Similarly, among those who had medium knowledge level, 33.9 % had medium adherence to medications level, 41.9 % had poor self-care behaviour and 38.7 % had poor glycemic control. Those respondents who had high knowledge level of DM, 25 % had low level of adherence to medications, 43.5 % had poor self-care behaviour and 25 % had poor glycemic control level.

**Table 2:** Multinomial logistic regression analyses findings of factors associated with knowledge on diabetes among T2DM patients JUTH, 2014

Variable		Knowledge, n (%) *n* = 309			Low versus High		Medium versus High	
		Low (*n* = 139)	Medium (*n* = 62)	High (*n* = 108)	COR (95%CI)	AOR (95%CI)	COR (95%CI)	AOR (95%CI)
Sex	Male	89 (64)	38 (61.3)	62 (57.4)	1.3 (0.8, 2.2)	-	1.2 (0.6, 2.2)	-
	Female	50 (36)	24 (38.7)	46 (42.6)	1	-	1	-
Age	<40 years	5 (3.8)	2 (3.4)	5 (5.1)	0.5 (0.1, 1.9)	-	0.5 (0.1, 3.1)	-
	40-60 years	80 (61.1)	39 (66.1)	69 (70.4)	0.6 (0.3, 1.1)	-	0.8 (0.4, 1.6)	-
	>60 years Missing	46 (35.1) 8	18 (30.5) 4	24 (24.5) 10	1	-	1	-
Marital status	Married	111 (79.9)	52 (83.9)	88 (81.5)	1.1 (0.5, 2.4)	0.9 (0.3, 2.3)	1.2 (0.5, 3.1)	0.7 (0.2, 2.4)
	Single	12 (8.6)	3 (4.8)	6 (5.5)	1.8 (0.5, 5.9)	1.6 (0.4, 7)	1 (0.2, 5.2)	0.6 (0.1, 3.9)
	Widow/er	16 (11.5)	7 (11.3)	14 (13)	1	1	1	1
Level of Education	Illiterate	60 (43.2)	18 (29)	31 (28.7)	2.6 (1.3, 4.9)*	3.1 (1.03, 9.3)*	0.9 (0.4, 2.04)	0.4 (0.1, 1.7)
	Primary	51 (36.7)	21 (33.9)	40 (37)	1.6 (0.9, 3.2)	1.9 (0.8, 4.9)	0.8 (0.4, 1.8)	0.4 (0.1, 1.3)
	2^0^ and above	28 (20.1)	23 (37.1)	37 (34.3)	1	1	1	1
Occupation	Employed	28 (20.1)	16 (25.8)	28 (25.9)	0.8 (0.3, 1.8)	0.6 (0.2, 1.8)	2 (0.6, 7.1)	1.4 (0.3, 6.2)
	Unemployed	31 (22.3)	18 (29)	29 (26.9)	0.8 (0.3, 1.9)	0.5 (0.2, 1.4)	2.1 (0.6, 7.6)	3.3 (0.7, 16.1)
	Merchant	14 (10.1)	6 (9.7)	9 (8.3)	1.2 (0.4, 3.6)	0.7 (0.2, 2.7)	2.3 (0.5, 10.6)	3.8 (0.7, 22.2)
	Farmer	48 (34.5)	18 (29)	28 (25.9)	1.3 (0.6, 3.09)	0.5 (0.2, 1.6)	2.3 (0.6, 7.9)	2.4 (0.5, 12.1)
	Daily labor	18 (12.9)	4 (6.5)	14 (13)	1	1	1	1
Family/social support	Yes	85 (61.2)	29 (46.8)	61 (56.5)	1.2 (0.7, 2)	0.9 (0.5, 1.7)	0.6 (0.4, 1.3)	0.6 (0.3,1.3)
	No	54 (38.8)	33 (53.2)	47 (43.5)	1	1	1	1
DM Family history	Yes	38 (27.3)	9 (14.5)	29 (26.9)	1.03 (0.6, 1.8)	1.3 (0.6, 2.5)	0.5 (0.2, 1.06)	0.5 (0.2, 1.4)
	No	101 (72.7)	53 (85.5)	79 (73.1)	1	1	1	1
Glucometer at home	Yes	12 (8.6)	3 (4.8)	15 (13.9)	0.6 (0.3, 1.3)	0.5 (0.2, 1.3)	0.3 (0.09, 1.1)	0.3 (0.07, 1.3)
	No	127 (91.4)	59 (95.2)	93 (86.1)	1	1	1	1
Cigarette smoking	Yes	10 (7.2)	5 (8.1)	7 (6.5)	1.1 (0.4, 3)	-	1.2 (0.4, 4)	-
	No	129 (92.8)	57 (91.9)	101 (93.5)	1	-	1	-
Alcohol drinking	Yes No	7 (5) 132 (95)	3 (4.8) 59 (95.2)	7 (6.5) 101 (93.5)	0.8 (0.3, 2.2) 1	- -	0.7 (0.2, 2.9) 1	- -
*Khat* chewing status	Yes	32 (23)	19 (30.6)	26 (24.1)	0.9 (0.5, 1.7)	-	1.4 (0.7, 2.8)	-
	No	107 (77)	43 (69.4)	82 (75.9)	1	-	1	-
BMI in Kg/m^2^	<18	11 (7.9)	3 (4.8)	3 (2.8)	6.9 (1.5, 32)*	6.4 (1.2, 34.9)*	2.5 (0.4, 16)	3.5 (0.4, 30.1)
	18-25	78 (56.1)	33 (53.2)	50 (46.3)	2.9 (1.2, 7.4)*	2.5 (0.9, 7)	1.6 (0.6, 4.7)	2.6 (0.8, 8.9)
	25-30	42 (30.2)	20 (32.3)	40 (37)	1.9 (0.8, 5.2)	2.4 (0.8, 6.9)	1.3 (0.4, 3.7)	2.09 (0.6, 7.4)
	> = 30	8 (5.8)	6 (9.7)	15 (13.9)	1	1	1	1
Self-care behaviour	Good	61 (43.9)	36 (58.1)	61 (56.5)	0.6 (0.3, 1)	0.5 (0.3, 0.9)*	1.1 (0.6, 2)	0.9 (0.4, 2.09)
	Poor	78 (56.1)	26 (41.9)	47 (43.5)	1	1	1	1
Duration of DM in year	<5	73 (52.5)	41 (66.1)	31 (28.7)	4.7 (2.3, 9.5)*	4.2 (1.9, 9.5)*	9.5 (3.3, 27.1)*	9.8 (3.2, 30.2)*
	5-10	48 (34.5)	16 (25.8)	41 (38)	2.3 (1.2, 4.7)*	1.8 (0.8, 3.9)	2.8 (0.9, 8.4)	2.5 (0.8, 8.1)
	>10	18 (13)	5 (8.1)	36 (33.3	1	1	1	1
Glycemic control level	Good	100 (71.9)	38 (61.3)	81 (75)	1.2 (0.7, 2)	-	1.9 (0.9, 3.7)	0.9 (0.4, 2.09)
	Poor	39 (28.1)	24 (38.7)	27 (25)	1	-	1	1

* Statistically significant at *P*-value < = 0.05

*T2DM* type 2 diabetes Mellitus, *JUTH* Jimma University Teaching Hospital

Table 2 also presents results of the multinomial logistic regression analysis of factors associated with knowledge of diabetes. High level of knowledge of diabetes was the reference group. Illiterate respondents compared to those who attained higher secondary education were highly likely (AOR = 3.1, 95%CI: 1.03-9.3) to have low level of knowledge. The relative probability of having a low level of knowledge among respondents who had BMI below 18 kg/m^2^ was significantly higher (AOR = 6.4, 95%CI: 1.2-34.9) than those who had 30 kg/m^2^ and above. Duration of DM of less than five (<5) years was associated with both low level of knowledge (AOR = 4.2, 95%CI: 1.9-9.5) and medium level of knowledge of diabetes (AOR = 9.8, 95%CI: 3.2-30.2).

### Self-care behaviours toward diabetes

The overall prevalence of poor self-care behaviours toward DM was 49.1 % (95%CI: 43.5-54.7 %). Poor self-care behaviours was statistically different by level of education, family history of DM, adherence to medications, having glucometer at home and history of alcohol consumption. Table 3 presents binary logistic regression results of factors independently associated with poor self-care behaviours.

**Table 3:** Factors independently associated with poor self-care behaviours among T2DM patients JUTH, 2014

Variables	Self-care behaviour (*n* = 309)			COR (95%CI)	AOR (95 % CI)
		Good n (%)	Poor n (%)		
Education	Illiterate	18 (20.7)	69 (79.3)	2.7 (1.5, 4.9)	3.1(1.7, 5.8)*
	Primary	10 (45.5)	12 (54.5)	1.8 (1, 3.1)	1.9(1.1, 3.6)*
	2^0^ and above	31 (27.7)	81 (72.3)	1	1
Family history	Yes	31 (19.7)	45 (29.6)	0.5 (0.3, 0.9)	0.5 (0.3, 0.9)*
	No	126 (80.3)	107 (70.4)	1	1
Adherence to medications	High	62 (53.9)	53 (46.1)	0.5 (0.3, 0.9)	0.6 (0.3, 1.1)
	Medium	68 (58.1)	49 (41.9)	0.4 (0.2, 0.7)	0.4 (0.3, 0.8)*
	Low	28 (36.4)	49 (63.6)	1	1
Have glucometer at home	Yes	12 (40)	18 (60)	1.7 (0.8, 3.6)	2.5 (1.1, 5.8)*
	No	146 (52.3)	133 (47.7)	1	1
History of alcohol drinking	Yes	4 (23.5)	13 (76.5)	3.6 (1.2, 11.4)	4.6 (1.3, 15.7)*
	No	154 (52.7)	138 (47.3)	1	1

* Statistically significant at *P*-value < = 0.05

*T2DM*, type 2 diabetes Mellitus, *JUTH* Jimma University Teaching Hospital

Respondents with lower educational level were likely to have poor self-care behaviour than those with higher secondary education (AOR = 3.1, 95%CI: 1.7, 5.8). Having previous family history of DM was found to be protective against poor self-care behaviours (AOR = 0.5, 9%CI: 0.3-0.9). Compared to respondents with low level of adherence to medications, those with medium level were 60 % less (AOR = 0.4, 95%CI: 0.3-0.8) likely to have poor self-care behaviours. Interestingly, respondents with glucometers at home were 2.5 times more likely to have poor self-care behaviours than those who did not have (AOR = 2.5, 95%CI: 1.1-5.8) glucometers at home. Compared to respondents who did not drink alcohol, those who had history of alcohol consumption were highly likely to have poor self-care behaviours (AOR = 4.6, 95%CI: 1.3-15.7).

### Level of anti-diabetic adherence to medications

Table 4 presents levels of adherence to medications by demographic and clinical characteristics. Different levels of adherence to medications were as follows: 24.9 % had low, 37.9 % had medium and 37.2 % had high level of adherence to medications. Of respondents with low level of adherence to medications, 55.8 % had low level of knowledge of diabetes, 63.6 % had poor self-care behaviours and 16.9 % had poor glycemic control level. Similarly, among those who had medium level of adherence to medications, 17.9 % had medium level of knowledge on diabetes, 41.9 % had poor self-care behaviours and 21.4 % had poor glycemic control. Those respondents who had high level of adherence to medications, 35.7 % had high level of diabetes knowledge, 53.9 % had good self-care behaviours and 54.8 % had good glycemic control level.

**Table 4:** Multinomial logistic regression analyses findings of factors associated with adherence to medications among T2DM patients JUTH, 2014

Variable		Adherence to medications, n (%)			Low versus High		Medium versus High	
		Low (*n* = 77)	Medium (*n* = 117)	High (*n* = 115)	COR (95%CI)	AOR (95%CI)	COR (95%CI)	AOR (95%CI)
Sex	Male	52 (67.5)	70 (59.8)	67 (58.3)	1.5 (0.8, 2.7)	1.5 (0.6, 3.8)	1.1 (0.6, 1.8)	1.5 (0.7, 3.4)
	Female	25 (32.5)	47 (40.2)	48 (41.7)	1	1	1	1
Age	<40 years	3 (4.1)	7 (6.4)	2 (1.9)	1.8 (0.3, 11.7)	-	4.6 (0.9, 23.9)	-
	40-60 years	42 (57.5)	77 (70)	69 (65.7)	0.7 (0.4, 1.4)	-	1.5 (0.8, 2.7)	-
	>60 years Missing	28 (38.4) 4	26 (23.6) 7	34 (32.4) 10	1	-	1	-
Marital status	Married	66 (85.7)	95 (81.2)	90 (78.3)	1.7 (0.6, 4.3)	-	1.2 (0.6, 2.6)	-
	Single	4 (5.2)	8 (6.8)	9 (7.8)	1.02 (0.2, 4.4)	-	1.02 (0.3, 3.4)	-
	Widow/er	7 (9.1)	14 (12)	16 (13.9)	1	-	1	-
Level of Education	Illiterate	29 (37.7)	47 (40.2)	33 (28.7)	1.9 (0.9, 4.1)	2.03 (0.6, 6.8)	1.9 (0.9, 3.6)	1.01 (0.4, 2.9)
	Primary	30 (39)	40 (34.2)	42 (36.5)	1.6 (0.8, 3.3)	1.3 (0.5, 3.8)	1.3 (0.7, 3.3)	0.7 (0.3, 1.7)
	2^0^ and above	18 (23.3)	30 (25.6)	40 (34.8)	1	1	1	1
Occupation	Employed	20 (26)	25 (21.4)	27 (23.5)	2.3 (0.8, 6.5)	2.9 (0.9, 9.7)	2.9 (1.06, 7.9)*	3.1 (1.01, 9.7)*
	Unemployed	15 (19.5)	33 (28.2)	30 (26.1)	1.6 (0.6, 4.5)	2.7 (0.7, 10.8)	3.5 (1.3, 9.3)*	8.2 (2.2, 30.4)*
	Merchant	13 (16.9)	9 (7.7)	7 (6.1)	5.8 (1.7, 20.4)*	6.8 (1.6, 28.8)*	4 (1.09, 14.9)*	5.3 (1.2, 22.8)*
	Farmer	22 (28.6)	43 (36.7)	29 (25.2)	2.4 (0.9, 6.6)	1.6 (0.4, 5.8)	4.7 (1.8, 12.3)*	3.8 (1.1, 12.8)*
	Daily labor	7 (9)	7 (6)	22 (19.1)	1	1	1	1
Family/social support	Yes	46 (59.7)	69 (59)	60 (52.2)	1.4 (0.8, 2.4)	-	1.3 (0.8, 2.2)	-
	No	31 (40.3)	48 (41)	55 (47.8)	1	-	1	-
DM Family history	Yes	18 (23.4)	34 (29.1)	24 (20.9)	1.2 (0.6, 2.3)	1.1 (0.5, 2.6)	1.6 (0.9, 2.8)	1.6 (0.8, 3.2)
	No	59 (76.6)	83 (70.9)	91 (79.1)	1	1	1	1
Glucometer at home	Yes	8 (10.4)	13 (11.1)	9 (7.8)	1.4 (0.5, 3.7)	-	1.4 (0.6, 3.6)	-
	No	69 (89.6)	104 (88.9)	106 (92.2)	1	-	1	-
Cigarette smoking	Yes	10 (13)	4 (3.4)	8 (7)	1.9 (0.8, 5.3)	2.3 (0.6, 8.6)	0.5 (0.1, 1.6)	0.4 (0.1, 1.6)
	No	67 (87)	113 (96.6)	107 (93)	1	1	1	1
Alcohol drinking	Yes No	8 (10.4) 69 (89.6)	5 (4.3) 112 (95.7)	4 (3.5) 111 (96.5)	3.2 (0.9, 11.1) 1	2.1 (0.5, 9.2) 1	1.2 (0.3, 4.7) 1	1.8 (0.4, 7.9) 1
*Khat* chewing status	Yes	18 (23.4)	38 (32.5)	21 (18.3)	1.4 (0.7, 2.8)	0.8 (0.4, 2.1)	2.2 (1.2, 3.9)*	1.9 (0.9, 3.9)
	No	59 (76.6)	79 (67.5)	94 (81.7)	1	1	1	1
BMI in Kg/m^2^	<18	7 (9.1)	6 (5.1)	4 (3.5)	5.6 (1.2, 27.4)*	4.9 (0.8, 31)	3 (0.7, 13.8)	2.9 (0.6, 16.3)
	18-25	46 (59.7)	69 (59)	46 (40)	3.2 (1.1, 9.5)*	2.6 (0.7, 9.1)	3 (1.2, 7.6)*	3.4 (1.2, 9.9)*
	25-30	19 (24.7)	34 (29.1)	49 (42.6)	1.2 (0.4, 3.9)	1.3 (0.3, 4.6)	1.4 (0.5, 3.6)	1.7 (0.6, 4.9)
	> = 30	5 (6.5)	8 (6.8)	16 (13.9)	1	1	1	1
Knowledge	High Medium	27 (35.1) 7 (9.1)	40 (34.2) 21 (17.9)	41 (35.7) 34 (29.6)	1.6 (0.9, 3.1) 0.3 (0.1, 0.8)*	1.1 (0.5, 2.4) 0.2 (0.1, 0.6)*	1.4 (0.8, 2.6) 0.6 (0.3, 1.3)	1.4 (0.7, 2.9) 0.5 (0.2, 1.2)
Self-care	Low	43 (55.8)	56 (47.9)	40 (34.8)	1	1	1	1
	Good	28 (36.4)	68 (58.1)	62 (53.9)	0.5 (0.3, 0.9)*	0.7 (0.3, 1.3)	1.2 (0.7, 1.9)	1.7 (0.9, 3.1)
	Poor	49 (63.6)	49 (41.9)	53 (46.1)	1	1	1	1
Duration of DM in year	<5	43 (55.8)	54 (46.2)	48 (41.7)	1.7 (0.8, 3.7)	2.1 (0.8, 5.6)	1.2 (0.6, 2.5)	1.02 (0.4, 2.4)
	5-10	21 (27.3)	41 (35)	43 (37.4)	0.9 (0.4, 2.2)	0.9 (0.4, 2.5)	1.04 (0.5, 2.1)	1.01 (0.4, 2.3)
	>10	13 (16.9)	22 (18.8)	24 (20.9)	1	1	1	1
Glycemic control level	Good	64 (83.1)	92 (78.6)	63 (54.8)	4.1 (2.02, 8.2)*	3.3 (1.5, 7.2)*	3.04 (1.7, 5.4)*	2.8 (1.5, 5.3)*
	Poor	13 (16.9)	25 (21.4)	52 (45.2)	1	1	1	1

*T2DM* type 2 diabetes mellitus, *JUTH* Jimma University Teaching Hospital, *COR* crude odds ratio, *AOR* adjusted odds ratio

*Statistically significant at *P*-value < = 0.05

Table 4 presents results of the multinomial logistic regression analysis of factors associated with adherence to medications. High level of adherence to medications was the reference group. Farmers compared to daily labourers were highly likely (AOR = 6.8, 95%CI: 1.6-28.8) to have low level of adherence to medications. Respondents with medium level of knowledge of diabetes were 80 % less (AOR = 0.2, 95%CI: 0.1-0.6) likely to have low level of adherence to medications. The relative probability of having a low level of adherence to medications among respondents who had good glycaemic control level was higher (AOR = 3.3, 95%CI: 1.5-7.2) than those who had poor glycaemic control level. The relative probability of having a medium level of adherence to medications among respondents who had BMI between 18 and 25 kg/m^2^ was significantly higher (AOR = 3.4, 95%CI: 1.2-9.9) than those who had 30 kg/m^2^ and above. Good glycaemic control level was significantly associated with medium level of adherence to medications (AOR = 2.8, 95%CI: 1.5-5.3).

## Discussion

Diabetes is a chronic condition with many complications, and its management would need sufficient levels of knowledge, self-care behaviours and adherence to medications [17, 24]. For effective management and in order to have good glycemic control, patients need to have adequate levels of knowledge of diabetes regarding self-care, a concept that can foster adherence to medications, good dietary pattern and physical activity [11, 25]. Very few studies have addressed the importance of knowledge, self-care behaviours and adherence to medications among diabetic patients in resource poor countries including Ethiopia [8, 15].

The findings of the current study conform with results from Asian and African studies that have revealed low level of knowledge of diabetes among participants, who also demonstrated to have poor self-care behaviours and poor adherence to medications [14, 16, 26–28]. However, studies from elsewhere have also reported contrasting findings [25, 29]. We argue that, these differences in findings could be due to differences in study populations as wells as the type of tools used to measures these outcomes. Consistent with the current study’s findings, knowledge has a significant effect on self-care behaviours and adherence to medications [30, 31]. As such, these findings inform of the necessity to have consistent diabetic education to address issues related to self-care behaviours and adherence to medications as both are the most cost effective management strategies for DM complications [32]. As the diabetic education alone would not be sufficient for sustained control, comprehensive and effective strategies comprising actions to enforce self-care behaviours such as good dietary patterns, regular physical activity, self-glycemic control and foot care [33] should be designed.

Respondents aged between 40 and 60 years were less likely to have low knowledge level than those in age group older than 60 years. This indicates that older people were at a higher risk and thus there would be a need to develop targeted programs to address inequity that existed between age groups. A significant difference in low level of knowledge of diabetes was observed among illiterates than those who attained higher secondary education. This is not surprising as knowledge is gained through education. This finding was consistent with other studies from United Arab Emirates (UAE) [16] and Bangladesh [14]. Lower education status could end up with low self-management behaviours, lower self-efficacy and lower continuity of care. Thus, as recommendations, measures to improve literacy level would be cost effective to reduce diabetic morbidity and mortality [12, 34]. Lower BMI and short duration of diabetes (less than five years) were significantly associated with level of knowledge and this was supported by findings from Nigeria [35] to UAE [16].

Almost half (49.1 %) of the respondents had poor self-care behaviours toward DM. Although these figures are lower than the previously reported elsewhere including in North Ethiopia (59 %) [15], East Ethiopia (60.8 %) [31] and Kenya (63.2 %), [28], the magnitude of diabetes in the current study society still denotes diabetes as a significant public health problem. The hypothesise that this variation could be due to the types instruments used in different studies or duration of patients on treatment. It well known that a well instituted diabetes self-care plan lowers glycosylated hemoglobin levels- an indicator that can be used to monitor diabetes [36].

Lower educational level and poor adherence to medications were among the predictors of poor self-care behaviours. This is similar to findings of the previous studies [31, 37, 38], and shows how diabetes education and its application is indispensable for diabetic management [32, 33]. Having family history of DM was found to be protective against poor self-care behaviours, a finding which is not dissimilar with findings of the previous studies [25, 39, 40]. It would be plausible to argue that diabetic patients would share their knowledge, and experiences with families members [25], information that could be used by the newly diagnosed members to improve their conditions through effective self-care behaviours.

The current study also revealed that respondents who had glucometer at home were 2.5 times more likely to have poor self-care behaviours than those who did not. This could be due to the fact that the majority (nearly 90 %) of the respondents had not been counseled on how to measure self-glucose level using glucometer. Besides, more than 90 % of respondents did not Self-Monitoring Blood Glucose (SMBG) service at home either due to lack of knowledge or scarcity of consumables. Consistent with findings from California [41] to United Kingdom [42], the relative probability of having poor self-care behaviours among respondents who had history of alcohol drinking was also significantly higher than those who did not drink. This is a clue for the inclusion of brief interventions strategy for alcohol in diabetes care.

This study reported that one fourth and two fifth of the respondents had low and medium level of adherence to diabetic medications respectively, the findings which were lower than a study from France [10]. The differences could be explained due to financial problem, management of side effect of the drugs, health care providers’ approach during diabetic education and counseling, and general quality of care for diabetic services in Ethiopia [8]. Furthermore, individuals with low socio-economic status have limited access to education, information and transportation, which are necessary drivers to required necessary services including medications.

In the current study, farmers compared to daily labours were high likely to have lower level of adherence to medications. This could be related with level of education as it was obvious that the majority of the farmers in this study were illiterate compared to daily labourers. In addition, other plausible factors including poor access to health care and allocating less time for self-care management could be barriers to adherence to medications. In contrast to other studies, [13, 43–45], findings from this study revealed that good glycaemic control level was significantly associated with low and medium level of adherence to medications. We hypothesise that these findings could be related to protection provided by participants life styles including being laborers where they would endure significant incidental physical activity and having dietary patterns that are plant based. However, these findings need further exploration.

This study had several limitations which were worth noting. The institutional based nature of the study might not infer for other diabetic patients. Similarly, the nature of cross-sectional study design does not indicate temporal relationship or causality. Self-report of adherence to medications could also be affected by recall bias. Moreover, selection bias could also have been introduced because patients who are under regular follow-up by the university clinic are likely to be receiving better care and support than those in the lower level clinics. We were unable to use HbA1c, a more accurate than FBG measurement to evaluate glycemic control due to inaccessibility and high cost of the measurement in our country. It is not routinely done as part of the standard care in the study hospital. This limitation is not only affecting our study, but also is a significant challenge for diabetes control in the country as a whole without access to Hb1Ac measurement. Finally, a psychometric study for the three tools: The Diabetes Knowledge Test (DKT), Expanded Version of the Summary of Diabetes Self-Care Activities (SDSCA) and Morisky scale was not conducted.

## Conclusion

In summary, the findings from the current study revealed that a significant number of diabetic patients had low level of knowledge, poor self-care behaviours and low level of adherence to medications. These findings suggest the need to work on integrated interventional management on diabetic knowledge, self-care behaviours and adherence to medications. Education, awareness creation and implementation of good self-care behaviours could be improved as cross cutting interventions. We recommend the inclusion of brief interventions strategy for alcohol in diabetes care. It has been reported that brief interventions to reduce at-risk drinking has the potential to improve diabetic medication adherence and treatment outcome [46]. We also recommend further population based research to explore specific factors such as the association found in this study that indicated that low level of adherence to medications was associated with good control of glycaemia. Where possible, psychometric studies should be conducted as tools to assess diabetic knowledge, self-care behavior and medication adherence.

## Abbreviations

BMI, body mass index; DM, diabetes mellitus; DKT, diabetes knowledge test; FBG, fasting blood glucose; HbA1c, glycosylated hemoglobin; IDFA, International Diabetes Federation Atlas; JUTH, Jimma University Teaching Hospital; OHA, oral hypoglycemic agent; SMBG, self-monitoring blood glucose; SDSCA, summary of diabetes self-care activities; T2DM, type-2 diabetes mellitus.

## Additional file

Additional file 1: Tools for assessing diabetes related knowledge, self-care behaviours and adherence to medications among diabetic patients in Southwest Ethiopia, 2014. (DOCX 110 kb)
