# Supplementary material for: Diabetes related knowledge, self-care behaviours and adherence to medications among diabetic patients in Southwest Ethiopia: a cross-sectional survey
Source: BMC Endocr Disord. 2016 May 31;16:28. doi: 10.1186/s12902-016-0114-x (PMC4933997; doi:10.1186/s12902-016-0114-x)
Supplement: Additional file 1: — Tools for assessing diabetes related knowledge, self-care behaviours and adherence to medications among diabetic patients in Southwest Ethiopia, 2014. (DOCX 110 kb) [file 12902_2016_114_MOESM1_ESM.docx]

**Questionnaire**

A structured questionnaire prepared to assess diabetes knowledge, self-care behaviors adherence and poor glycemic control among patients with type 2DM. Jan 30 to Feb 28. Each page will filled by data collectors according to the patient’s response and review the card. And then circle the correct response.

**PART ONE**

**Socio-demography characteristics**

1. Cared number _____________
2. Age_____________________
3. Height (cm)_______________
4. Weight (kg) _______________
5. Sex

A, Male B, Female

1. Current marital status

A, Single B, Married C, Divorce D, Widow/er

1. Level of education

A, Illiterate C, primary school [1-8] E, college/university

B, Informal education D, secondary school [9-12]

1. Monthly income (birr)_____________
2. Occupation/ Employment

A, Employed B, Unemployment C, Merchant D, Farmer E, other____

1. Family / social support

A, Yes B, No

1. Ethnicity

A, Oromo B, Amhara C, Dawero D, Tigre E, Other_________

1. Religion

A, Orthodox B, Muslim C, Protestant D, Catholic E, Others__________

**PART TWO**

**The following question assess the patient health status & medication related variable**

1. Duration of the diabetes, since diagnosis, in year________________
2. Do you have family history of diabetes?

A, Yes B, No

1. How many medications have taken per day? _____________
2. Do you have Diabetic related complication? If yes, which one of the following

A, neuropathy B, nephropathy C, retinopathy D, Other_______________

1. Currently, do you have your own glucometer at home?

A, Yes B, No

If yes, how many times measure your glucose level per day? __________

1. Cigarette smoking status

A, Smoker B, Non-smoker C, Ex-smoker

If you smoke, how many cigarettes did you smoke on an average day?

Number of cigarette:_____________

1. Alcohol drinking status?

A, drinker B, Non-drinker C, Ex-drinker

1. Have you ever been hospitalized due to Diabetic related problem?

A, Yes B, No

If yes, how many times per year? _____________

Cause of hospitalization________________

**PART THREE**

Assessing determinants of non-adherence will be calculated using Morisky 8-Item Medication Adherence Questionnaire

1. Do you sometimes forget to take your medicine?

A, YES B, NO

1. People sometimes miss taking their medicines for reasons other than forgetting. Thinking over the past 2 weeks, Were there any days when you did not take your medicine in the past two weeks?

A, YES B, NO

1. Have you ever cut back or stopped taking your medicine without telling your doctor because you felt worse when you took it?

A, YES B, NO

1. When you travel or leave home, do you sometimes forget to bring along your medicine?

A, YES B, NO

1. Did you take all your medicines yesterday?

A, YES B, NO

1. When you feel like your symptoms are under control, do you sometimes stop taking your medicine?

A, YES B, NO

1. Taking medicine every day is a real inconvenience for some people; do you ever feel hassled about sticking to your treatment plan?

A, YES B, NO

1. How often do you have difficulty remembering to take all your medicine?

A, Never/ Rarely C, Sometimes E, All the time

B. Once in a while D, Usually

**PART FOUR**

For assessing patient knowledge related to diabetes will use Diabetes Knowledge Questionnaire (DKQ). It consists of 23 questions concerning general patient knowledge of diabetes self -care practices. The entire questionnaire can be administered to patients who use insulin; But only the first 14 questions apply to patient s who do not use this agent.

401. The diabetes diet is:

a. The way most Ethiopian people eat b. A healthy diet for most people

c. Too high in carbohydrate for most people d. Too high in protein for most people

402. Which of the following is highest in carbohydrate?

a, Baked chicken b. Ergo

c. Baked potato d. Peanut butter

403. Which of the following is highest in fat?

a. Milk b. Orange juice

c. Corn d. Honey

404. Which of the following is a “sugar free food”?

a. Any unsweetened food b. Any dietetic food

c. Any food that says “sugar free” on the label d. Any food that has less calories

Per-serving

405. Fast blood sugar is a test that is a measure of your blood glucose level for the past:

a. Day b. Week

c. 6-10weeks d. 6 months

406. Which is the best method for testing blood glucose?

a. Urine testing b. Blood testing c. Both are equally good

407. What effect does unsweetened fruit juice have on blood glucose?

a. Lowers it b. Raises it c. Has no effect

408. Which should not be used to treat low blood glucose?

a. 3 hard candies b. 1/2 cup orange juice

c. 1cup soft drink d.1cup milk

409. For a person in good control, what effect does exercise have on blood glucose?

a. Lowers it b. Raises it c. Has no effect

410. Infection is likely to cause:

a. An increase in blood glucose b. Decrease in blood glucose

c. No change in blood glucose

411. The best way to take care of your feet is to:

a. Look at and wash them each day b. Massage them with alcohol each day

c. Soak them for one hour each day d. Buy shoes a size larger than usual

412. Eating foods lower in fat decreases your risk for:

a. Nerve disease b. Kidney disease

c. Heart disease d. Eye disease

413. Numbness and tingling may be symptoms of:

a. Kidney disease b. Nerve disease

c. Eye disease d. liver disease

414. Which of the following is usually not associated with diabetes?

a. Vision problems b. Kidney problems

c. Nerve problems d. Lung problems

**II- Do you have taken insulin injection?**

A, Yes B, No

If yes, the remaining questioners will be filled

415. Signs of hyperglycemia include:

a. Shakiness b. Sweating

c. Vomiting d. Low blood glucose

416. If you are sick with the flu, which of the following changes should you make?

a. Take less insulin b. Drink less liquids

c. Eat more proteins foods d. Test for glucose more often

417. If you have taken insulin Lente, you are most likely to have an insulin action in:

a. 1-3hours b. 6-12 hours

c. 12-15hours d. More than 15 hours

418. You realize just before lunch time that you forgot to take your insulin before breakfast. What should you do now?

a. Skip lunch to lower your blood glucose

b. Take the insulin that you usually take at breakfast

c. Take twice as much insulin as you usually take at breakfast

d. Check your blood glucose level to decide how much insulin to take

419. If you are beginning to have an insulin reaction, you should:

a. Exercise b. Lie down and rest

c. Drink some juice d. Take regular insulin

420. Low blood glucose may be caused by:

a. Too much insulin b. Too little insulin

c. Too much food d. Too little exercise

421. If you take your morning insulin but skip breakfast your blood glucose level will usually:

a. Increase b. Decrease c. Remain the same

422. High blood glucose may be caused by:

a. Not enough insulin b. Skipping meals

c. Delaying your snack d. Large ketones in your urine

423. Which one of the following will most likely cause an insulin reaction?

a. Heavy exercise b. Infection

c. Overeating d. Not taking your insulin

**PART FIVE**

The following questions will assess the patient self-care behaviors by using Expanded Version of the **Summary of Diabetes Self-Care Activities (SDSCA).** So the following questions will ask you about your diabetes self-care activities during the past 7 days. If you were sick during the past 7 days, please think back to the last 7 days that you were not sick.

**Self-Care Recommendations**

1A. which of the following has your health care team (doctor, nurse, dietitian, or diabetes educator) advised you to do? Please check all that apply:

a. Follow a low-fat eating plan

b. Follow a complex carbohydrate diet

c. Reduce the number of calories you eat to lose weight

d. Eat lots of food high in dietary fiber

e. Eat lots (at least 5 servings per day) of fruits and vegetables

f. Eat very few sweets (for example: desserts, non-diet sodas, candy bars)

g. Other (specify):_____________________________________________

h. I have not been given any advice about my diet by my health care team.

2A.Which of the following has your health care team (doctor, nurse, dietitian or diabetes educator) advised you to do? Please check all that apply:

a. Get low level exercise (such as walk-ing) on a daily basis.

b. Exercise continuously for a least 20 minutes at least 3 times a week.

c. Fit exercise into your daily routine (for example, take stairs instead of elevators, park a block away and walk, etc.)

d. Engage in a specific amount, type, duration and level of exercise.

e. Other (specify): _________________________________________________________

f. I have not been given any advice about exercise by my health care team.

3A. which of the following has your health care team (doctor, nurse, dietitian, or diabetes educator) advised you to do? Please check all that apply:

a. Test your blood sugar using a drop of blood from your finger and a color chart.

b. Test your blood sugar using a machine to read the results.

c. Test your urine for sugar.

d. Other (specify):_________________________________________________________

e. I have not been given any advice either about testing my blood or urine sugar level by my health care team

4A. which of the following medications for your diabetes has your doctor pre-scribed? Please check all that apply.

a. An insulin shot 1 or 2 times a day.

b. An insulin shot 3 or more times a day.

c. Diabetes pills to control my blood sugar level.

d .Other (specify): ____________________________________________________

e. I have not been prescribed either insulin or pills for my diabetes

**Diet**

How many of the last SEVEN DAYS have you followed a healthful eating plan?

**0 1 2 3 4 5 6 7**

On average, over the past month, how many DAYS PER WEEK have you followed

You’re eating plan?

**0 1 2 3 4 5 6 7**

On how many of the last SEVEN DAYS did you eat five or more servings of fruits and vegetables?

**0 1 2 3 4 5 6 7**

On how many of the last SEVEN DAYS did you eat high fat foods such as red meat or

Full fat dairy products?

**0 1 2 3 4 5 6 7**

On how many of the last SEVEN DAYS did you space carbohydrates evenly through the day?

**0 1 2 3 4 5 6 7**

**Exercise**

On how many of the last SEVEN DAYS did you participate in at least 30 minutes of physical activity? (Total minutes of continuous activity including walking)

**0 1 2 3 4 5 6 7**

On how many of the last SEVEN DAYS did you participate in a specific exercise session (such as swimming, walking, biking) other than what you do around the house or as part of your work?

**0 1 2 3 4 5 6 7**

**Medications**

On how many of the last SEVEN DAYS, did you take your recom-mended diabetes medication?

**0 1 2 3 4 5 6 7**

**Blood Sugar Testing**

On how many of the last SEVEN DAYS did you test your blood sugar?

**0 1 2 3 4 5 6 7**

On how many of the last SEVEN DAYS did you test your blood sugar the number of times recommended by your health care provider?

**0 1 2 3 4 5 6 7**

**Foot Care**

On how many of the last SEVEN DAYS did you check your feet?

**0 1 2 3 4 5 6 7**

On how many of the last SEVEN DAYS did you inspect the inside of your shoes?

**0 1 2 3 4 5 6 7**

9A. on how many of the last SEVEN DAYS did you wash your feet?

**0 1 2 3 4 5 6 7**

10A. on how many of the last SEVEN DAYS did you soak your feet?

**0 1 2 3 4 5 6 7**

11A. on how many of the last SEVEN DAYS did you dry between your toes after washing?

**0 1 2 3 4 5 6 7**

**Smoking**

12A. At your last doctor’s visit, did anyone ask about your smoking status?

**0. No 1. Yes**

13A. If you smoke, at your last doctor’s visit, did anyone counsel you about stopping smoking or offer to refer you to a stop-smoking program?

0. No 1. Yes 2. Do not smoke.

14A.When did you last smoke a cigarette?

More than two years ago, or never smoked

One to two years ago

Four to twelve months ago

One to three months ago

Within the last month

Today

**PART SIX**

Data abstractions format will be filled from patient chart about the Patient Blood glucose level, lipid profile (mg/dl) and type of diabetic medication will be reviewed from the patient chart, retrospectively

| Lab test | Date | | | |  |
| --- | --- | --- | --- | --- | --- |
|  | Visit-1 | Visit-2 | Visit-3 | Visit-4 | Visit-5 |
| FBS |  |  |  |  |  |
| RBS |  |  |  |  |  |
| cholesterol |  |  |  |  |  |
| triglyceride |  |  |  |  |  |
| Serum LDL |  |  |  |  |  |
| Serum HDL |  |  |  |  |  |
| Type of Anti-diabetics drugs that you will take | A, oral hypoglycemia [OH]  B, insulin injection  C, OH + insulin | | | | |
